# Production of a Cloned Offspring and CRISPR/Cas9 Genome Editing of Embryonic Fibroblasts in Cattle

**DOI:** 10.1134/S1607672921010099

**Published:** 2021-03-10

**Authors:** G. N. Singina, P. V. Sergiev, A. V. Lopukhov, M. P. Rubtsova, N. P. Taradajnic, N. V. Ravin, E. N. Shedova, T. E. Taradajnic, I. A. Polejaeva, A. V. Dozev, G. Brem, O. A. Dontsova, N. A. Zinovieva

**Affiliations:** 1 Ernst Federal Science Center for Animal Husbandry, Podolsk, Russia; 2 Institute of Functional Genomics, Moscow State University, Moscow, Russia; 3 Center of Life Sciences, Skolkovo Institute of Science and Technology, Skolkovo, Russia; 4 Faculty of Chemistry, Moscow State University, Moscow, Russia; 5 Research Center of Biotechnology, Moscow, Russia; 6 Department of Animal, Dairy and Veterinary Sciences, Utah State University, Logan, UT USA; 7 Department of Animal Breeding and Genetics, University of Veterinary Medicine, Vienna, Austria; 8 Belozersky Research Institute of Physico-Chemical Biology, Moscow State University, Moscow, Russia; 9 Shemyakin–Ovchinnikov Institute of Bioorganic Chemistry, Moscow, Russia

**Keywords:** *Bos taurus*, somatic cloning, gene editing, beta-lactoglobulin knock-out

## Abstract

Somatic Cell Nuclear Transfer (SCNT) technique was used to produce the first viable cloned cattle offspring in Russia. Whole-genome SNP genotyping confirmed that the cloned calf was identical to the fibroblast cell line that was used for SCNT. CRISPR/Cas9 approach was subsequently used to knock out genes for beta-lactoglobulin gene (PAEP) and the beta-lactoglobulin-like protein gene (*LOC100848610)* in the fibroblast cells. Gene editing (GE) efficiency was 4.4% for each of these genes. We successfully obtained single-cell-derived fibroblast colonies containing *PAEP* and *LOC100848610* knockouts, which will be used to produce beta-lactoglobulin-deficient cattle.

The gene editing (GE) technology in combination with the technology of somatic cell nuclear transfer (SCNT) has broad prospects for solving problems associated with creating new genotypes, including those with modified economically useful traits [1, 2]. In particular, obtaining cloned embryos using embryonic fibroblasts, in the genome of which the β-lactoglobulin gene *(BGL)* is inactivated, and their transplantation to recipient animals is expected to produce cows capable of producing milk with reduced allergenic properties [3]. The use of a CRISPR/Cas9-based system as a tool for editing the genome of somatic cells should be considered as the most effective and innovative approach that is increasingly being used in domestic animals [4, 5]. The SCNT method allows selection of mutant cells prior to the start of expensive animal experiments and ensures obtaining offspring with desired gene modifications. The attractiveness of the CRISPR/Cas9-based system is determined by its high efficiency, simplicity, and low labor intensity [6].

The goal of this study was to construct SCNT-embryos of cattle using embryonic fibroblasts as donors of nuclei, to assess their development to viable offspring, as well as to obtain a culture of individual fibroblasts with knocked-out β-lactoglobulin gene.

For SCNT, post mortem isolated oocyte–cumulus complexes (OCC, *n* = 1332) were matured in TC-199 medium supplemented with 10% fetal bovine serum, 10 μg/mL follicle-stimulating hormone (FSH), and 10 μg/mL luteinizing hormones (LH). After 20–24 h of maturation, the OCCs was treated with 0.1% hyaluronidase solution, cumulus cells were mechanically removed, and oocytes with the first polar body (PPT, *n* = 1088) were collected. Embryonic fibroblasts were cultured to a formed monolayer, inhibited by contact for 2 days, and prepared in the form of a suspension for subsequent transfer to an enucleated oocyte (Fig. 1a). To fuse oocytes and cells transferred into their perivitelline space, two successive DC pulses with a voltage of 35 V and a duration of 20 μs were used (once or twice in the absence of signs of cell fusion). The resulting cytohybrids (*n* = 422, 44.5% of the number of oocytes with PPT) were activated with ionomycin 2 h after fusion and cultured to the blastocyst stage (Fig. 1b). To assess the normality and viability of the obtained embryos, some of them (*n* = 81) were transplanted to recipients synchronized in the cycle, and the remaining blastocysts were used for cytological analysis of the state of the nuclear material (*n* = 16; average number of nuclei, 78.3). Heifers of breeding age were used as recipients in a spontaneous and synchronized cycle. Before transplantation, sacral epidural anesthesia was performed with a 2% novocaine solution. Embryo transfer (1–6 embryos per animal) was performed by a non-surgical method deep into the uterine horn.

**Fig. 1.**
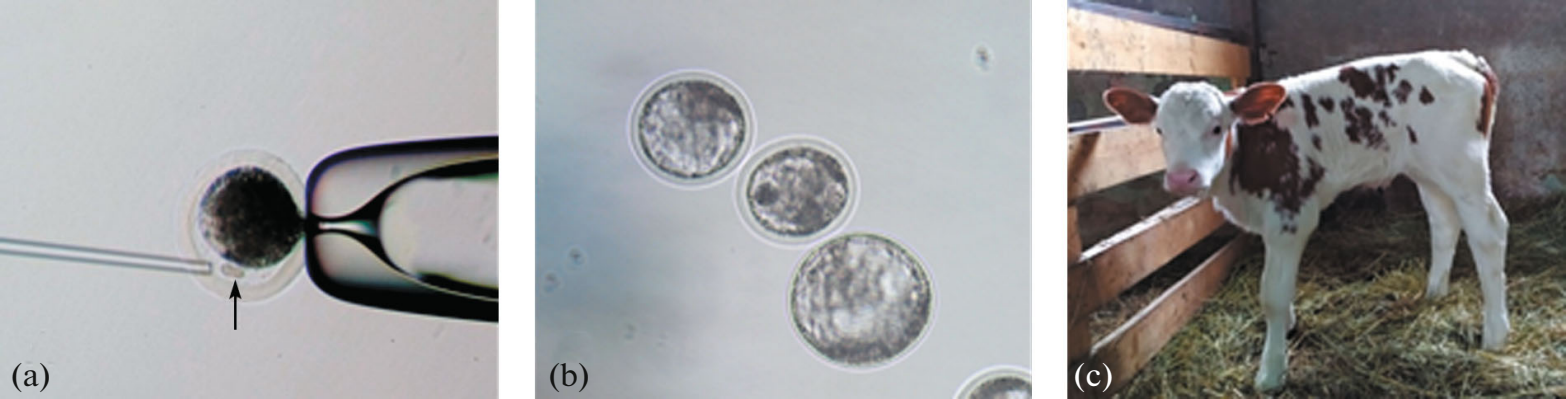
Photographs of (a) the procedure of somatic cell transfer (shown with the arrow) into the perivitelline space of an enucleated oocyte, (b) cloned bovine embryos used for transplantation to recipient animals, and (c) cloned calf (obtained in Russia for the first time).

It was found that, after activation, 64.5% (272/422) of cytohybrids formed 2-cell embryos and 23.0% (97/422) develop to the blastocyst stage. The proportion of pregnant animals after transplantation of the cloned blastocysts to 31 recipients was 43.8% (14/31), and the proportion of live births was 3.3% (1/31) (Fig. 1c).

To assess the origin of the obtained calf, genome-wide SNP genotyping of the offspring and the cell line was performed using the Bovine GGP HD high-density DNA chip (Neogen/Illumina Inc., United States). To estimate the degree of similarity, the IBS ​​(identity by state) distance values were calculated using the PLINK 1.9 software. IBS distance values ​​for the calve–cell line pairs were 1000, which confirms the identity of the genotype of the obtained calf and the cell line (for comparison, the IBS distances between unrelated animals of the Simmental breed vary in the range 0.731–0.763).

Taking into account the fact that, in the bovine genome, the beta-lactoglobulin (*BLG*) gene is duplicated and represented by two closely related paralogs, *BLG* (*PAEP)* itself and *BLG*-like gene (*LOC100848610*), we selected a strategy involving inactivation of both paralogous genes (Fig. 2).

**Fig. 2.**
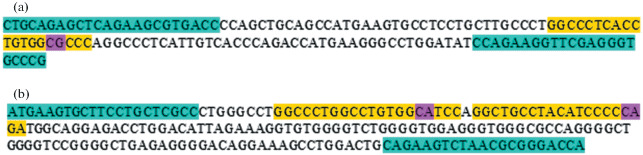
Scheme of the regions of (a) *PAEP* (*BLG)* and (b) *LOC100848610* (BLG-like protein) genes in the *Bos taurus* genome, which were selected as target for gene editing. The hybridization regions of primers used for amplifying genomic fragments are shown in blue. The presumable site of DNA cleavages by the CRISPR/Cas9 system are shown in purple. The hybridization regions of guide RNAs are shown in yellow.

When creating a genetic construct based on CRISPR, the pX458 vector [7] was selected as the basis for inactivation of the *PAEP* and *LOC100848610* genes [7]. This vector contains the hybrid *Cas9* gene and the gene encoding the green fluorescent protein (*GFP*), the coding regions of which are separated from each other by the sequence encoding the P2A peptide. The pX458 plasmid was cut with the *Bbs*I restriction enzyme. Hybridized oligonucleotides containing guide RNA sequences were ligated from the plasmid backbone. After ligation and transformation of competent *E. coli* JM109 cells, the grown colonies were used to accumulate the biomass and isolate plasmids, which, on the basis of the confirmation of the successful cloning of the constructs by sequencing, were used for transfection of bovine fibroblasts. The transfected somatic cells carrying the plasmid encoding the components of the CRISPR/Cas9 system were separated from the untransfected cells using the high-throughput BD FACSAria III cell sorter.

High-throughput sequencing of the amplicons of the target regions of the *PAEP* and *LOC100848610* genes in the pre-sorted population of embryonic fibroblasts showed the presence, respectively, of 12 and 7.5% of mutant sequences containing deletions and insertions of nucleotides at the sites of putative breaks.

To obtain individual colonies of bovine GE fibroblasts, the early-passage fibroblasts were electroporated with a mixture of plasmids encoding Cas9 and gRNA, aimed at inactivating the *PAEP* and *LOC100848610* genes (Fig. 3a).

**Fig. 3.**
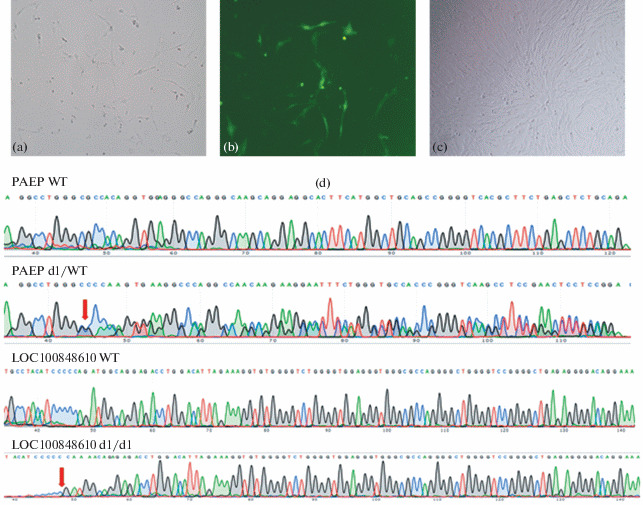
Micrographs of (a) the total pool of transfected *Bos taurus* fibroblasts 48 h after electroporation with a mixture of plasmids encoding Cas9 and gRNA, aimed at inactivating the *PAEP* and *LOC100848610* genes and (b) the cells expressing the genes of the CRISPR/Cas9 and GFP system components (green luminescence). (c) Culture of an individual colony of transfected cells. (d) Results of sequencing the amplicon of the *PAEP* gene fragment and the *LOC100848610* gene. Microinsertions and microdeletions in the region of gene cutting by the CRISPR/Cas9 system can be seen. Genotypes are signed. The sites of mutations are indicated with arrows.

After sorting (as described above, Fig. 3b), the total pool of the cells expressing the genes for components of the CRISPR/Cas9 system was grown for 2–3 days, after which the cells were seeded individually in 96-well plates and cultured until colonies were obtained (Fig. 3c) and formed 80–90% of the monolayer. The proportion of the formed colonies was 21.3% of the total number of cells (90/389). One part of each colony was frozen for possible further use, and the other part was used for DNA extraction and analysis for mutations. For this purpose, the *PAEP* and *LOC100848610* gene fragments containing the target regions were amplified, with subsequent Sanger sequencing (Fig. 3d). Knockout of *PAEP* and *LOC100848610* genes was established in 4 out of 90 obtained colonies of individual fibroblasts, which corresponds to a gene editing efficiency of 4.4%.

Thus, as a result of the study, the competence of the line of fetal bovine fibroblasts to develop to viable offspring was confirmed by the SCNT method; for the first time, using the CRISPR/Cas9 system, a line of embryonic fibroblasts with knockout of the *PAEP* and *LOC100848610* genes was obtained. The resulting cell line will be used to produce GE offspring of cattle with no beta-lactoglobulin synthesis.
